# POST-TRANSPLANT FUNCTIONAL STATUS TRAJECTORY AMONG ADULT KIDNEY TRANSPLANT RECIPIENTS

**DOI:** 10.1093/geroni/igz038.2921

**Published:** 2019-11-08

**Authors:** Nadia M Chu, Zhan Shi, Christine Haugen, Dorry Segev, Mara McAdams-DeMarco

**Affiliations:** 1 Department of Surgery, Johns Hopkins School of Medicine, Baltimore, Maryland, United States; 2 Department of Epidemiology, Johns Hopkins Bloomberg School of Public Health, Baltimore, Maryland, United States

## Abstract

Frailty and disabilities are highly prevalent among kidney transplant (KT) recipients, but are not routinely measured in KT recipients. The Karnofsky Performance Scale (KPS) is a clinically perceived measure used to evaluate patient’s ability to manage daily activities, but little is known about its post-KT trajectories and its relationship to frailty and disability in KT recipients. We leveraged a cohort of 159,992 adult KT recipients from SRTR (1/2005-6/2018) and a cohort of 1,106 adult KT recipients from a prospective cohort study on aging and KT with recorded KPS (range 10%-100% integers). In each separate cohort, we used mixed effects models to assess differences in trajectories of KPS post-KT. In 159,992 KT recipients in SRTR, the mean unadjusted KPS score was 88.34% (95%CI: 88.28%, 88.40%) and declined at a rate of -0.59%/year (95%CI: -0.61%, -0.57%) post-KT, such that by 2-years post-KT the average was 87.00% (95%CI: 86.94%, 87.05%). Age at KT was associated with steeper decline in KPS (p0.05). KPS is a measure of functional status distinct from frailty, ADL, IADL, and SPPB at KT admission that declines with older age post-KT. Older KT recipients should be monitored closely for declines in physical function, and potentially undergo prehabilitation to improve functional status post-KT.

